# Comparison of microbial diversity determined with the same variable tag sequence extracted from two different PCR amplicons

**DOI:** 10.1186/1471-2180-13-208

**Published:** 2013-09-14

**Authors:** Yan He, Ben-Jie Zhou, Guan-Hua Deng, Xiao-Tao Jiang, Hai Zhang, Hong-Wei Zhou

**Affiliations:** 1 Department of Environmental Health, School of Public Health and Tropical Medicine, Southern Medical University, Guangzhou, Guangdong 510515, China; 2 Department of Pharmacy, Zhujiang Hospital, Southern Medical University, Guangzhou 510282, Guangdong, China; 3 Network Center, Southern Medical University, Guangzhou, Guangdong, China

**Keywords:** Microbial diversity, V46 hypervariable region, V6 hypervariable region, Illumina, 16S rRNA gene, Meta-analysis

## Abstract

**Background:**

Deep sequencing of the variable region of 16S rRNA genes has become the predominant tool for studying microbial ecology. As sequencing datasets have accumulated, meta-analysis of sequences obtained with different variable 16S rRNA gene targets and by different sequencing methods has become an intriguing prospect that remains to be evaluated experimentally.

**Results:**

We amplified a group of fecal samples using both V4F-V6R and V6F-V6R primer sets, excised the same V6 fragment from the two sets of Illumina sequencing data, and compared the resulting data in terms of the α-diversity, β-diversity, and community structure. Principal component analysis (PCA) comparing the microbial community structures of different datasets, including those with simulated sequencing errors, was very reliable. Procrustes analysis showed a high degree of concordance between the different datasets for both abundance-weighted and binary Jaccard distances (P < 0.05), and a meta-analysis of individual datasets resulted in similar conclusions. The Shannon’s diversity index was consistent as well, with comparable values obtained for the different datasets and for the meta-analysis of different datasets. In contrast, richness estimators (OTU and Chao) varied significantly, and the meta-analysis of richness estimators was also biased. The community structures of the two datasets were obviously different and led to significant changes in the biomarkers identified by the LEfSe statistical tool.

**Conclusions:**

Our results suggest that beta-diversity analysis and Shannon’s diversity are relatively reliable for meta-analysis, while community structures and biomarkers are less consistent. These results should be useful for future meta-analyses of microbiomes from different data sources.

## Background

Determining 16S rRNA gene tag sequences using next generation sequencing (NGS) techniques, mainly the 454 and Illumina system platforms, has become a revolutionary tool in the field of microbiome research [1-4]. The major advantages of NGS methods are high-throughput capabilities and cost-effectiveness. Thousands of sequences per microbiome sample can be obtained easily, and hundreds to thousands of samples can be sequenced simultaneously [5]. However, the sequencing lengths obtained by NGS are shorter than those obtained by the Sanger sequencing method, and only part of the 16S rRNA gene spanning one or more of the nine hypervariable regions can be determined [4]. The first published study using NGS to study microbiomes determined the V6 tag of the 16S rRNA gene, and this region was short enough to be analyzed by the 454 Genome Sequencer 20 system at that time [6]. With the improvement of NGS techniques, sequencing lengths have grown to hundreds of bases per read, with even longer tags expected in the near future [5]. Although the short tag has proven useful for taxonomy assignment [7], longer tags may provide higher resolution for differentiating microbes and better taxonomy results. Until now, there has been no standard or best choice for choosing the variable regions, and researchers have historically determined diverse variable regions with varied sequencing lengths both within and across labs [3,4,8-10].

One of the major advantages of using DNA sequences to analyze microbiome diversity is that sequencing data obtained from different studies can be analyzed together, constituting a more cumulative approach than comparing DNA fingerprinting results [5]. However, if and how datasets from different sequencing projects can be combined for meta-analysis has not been evaluated because few studies have sequenced and compared actual microbiome samples processed by different experimental methods. One of the most straightforward ideas is to use the same variable region for different PCR amplicons and extract sequences of that specific region from different studies for direct comparison. Theoretically, the same tag region allows for a consistent clustering of operational taxonomic units (OTUs) and taxonomy assignment; therefore, subsequent parameters, including α- and β-diversities and community structures, can be analyzed. However, experimental conditions such as primer bias and sequencing quality might affect these analyses [11]. Until now, there have been no reports addressing this approach by amplifying real samples with different primers and extracting the same variable tag for direct comparison. In this study, we determined a total of 28 fecal microbiome samples from four individuals and amplified each sample independently with two primer sets (V4F-V6R and V6F-V6R). We analyzed the α-diversity, β-diversity, microbial community structure, and biomarkers and focused on the following two questions: First, do the results from the two datasets agree with one another? Second, can the two datasets be combined to produce reliable results? The present study provides useful information for evaluating the feasibility of meta-analysis for the study of microbiomes.

## Methods

### Ethical statement

This study was approved by the Ethical Committee of Southern Medical University, and all participants provided written informed consent.

### Sample processing and sequencing

Fecal samples were obtained from four individuals. For each individual, one sample was collected every two days for a period of two weeks. All of the samples were stored at -80°C until DNA extraction, and 200 mg of each sample was used for DNA extraction. DNA was extracted using the PowerSoil DNA kit (MoBio, USA) according to the manufacturer’s instructions. The high fidelity ExTaq cocktail (Takara, China) was used to amplify the 16S rRNA gene tags. Each DNA sample was amplified by 2 barcoded primer sets, one of which included the primers V4F 5′ GTGCCAGCMGCCGCGGTAA 3′ and V6R 5′ ACAGCCATGCANCACCT 3′, while the other included the primers V6F 5′ CNACGCGAAGAACCTTANC 3′ and V6R 5′ ACAGCCATGCANCACCT 3′. The PCR conditions included an initial denaturation step at 94°C for 2 min, 25 cycles of 94°C for 30 s, 52 ~ 59°C for 30 s and 72°C for 30 s, and a final extension at 72°C for 5 min. Each 25-μl reaction consisted of 2.5 μl of Takara 10× Ex Taq Buffer (Mg^2+^ free), 2 μl of dNTP Mix (2.5 mM), 1.5 μl of Mg^2+^ (25 mM), 0.25 μl of Takara Ex Taq DNA polymerase (2.5 units), 1 μl of template DNA, 0.5 μl of 10 μM barcode primer 967 F, 0.5 μl of 10 μM primer 1406R, and 16.75 μl of ddH_2_O. The two PCR products were sequenced independently in two sequencing batches at the Beijing Genomic Institute using paired-end sequencing with an Illumina HiSeq 2000 platform, and 101 bp were sequenced from each end. The sequences have been deposited in the sequence read archive (SRA) with accession number from ERS346316 to ERS346371.

### Sequence processing and analysis

We wrote a Perl script to separate tags according to their barcodes with the following steps: the primer region of each tag was first identified with no mismatches allowed; tags which failed to match primers were replaced by their reverse complements, and the primer region was identified again; the barcodes (region before the primer) and target V6 region (region after the primer) were stored for each tag; tags were separated according to their barcodes, and tags without any matching samples were discarded. For quality control purposes, no mismatches were allowed in the primer or barcode regions (see above). Furthermore, we removed tags with ambiguous bases (N) and screened potential chimeras with UCHIME (de novo mode, parameters set as follows: --minchunk 20 --xn 7 –noskipgaps 2 [12]. To unify the target region of the tags from the two primer sets, we extracted the V6 region of each tag by cutting 60 bp from the right end of the sequences from V6R primers (960 bp to 1,028 bp in E. coli). To avoid the effects of different sequencing depths, all samples were normalized to 5,000 sequences for subsequent analyses. We calculated the Good’s coverage of each sample at this depth. The formula used was $C=1-\frac{n}{N}$, where C is the Good’s coverage, n is the number of OTUs with only one tag per sample, and N is the number of all tags in that sample. TSC was used to cluster the tags into OTUs, with the similarity threshold set to 0.97 [13]. GAST was used to assign these sequences into taxa with the V6 database [7]. The α-diversity indices, including Chao, Ace, Shannon and observed OTUs, were calculated using the MOTHUR [14]. PCA was implemented using QIIME based on the Jaccard distance [15]. LEfSe was used to determine the biomarkers with LDA = 3 [16]. Statistical analysis was performed using SigmaPlot 12.0.

## Results and discussion

### Illumina paired-end sequencing results

In total, we determined 417,821 tags with the V4F-V6R primer set (an average of 14,992 tags per sample) and 756,514 tags with the V6F-V6R primer set (an average of 27,018 tags per sample). We discarded tags with mismatches in the primer regions, which reduced the total number of tags to 306,328 for the V4F-V6R dataset (an average of 10,940 tags per sample) and to 715,634 for the V6F-V6R dataset (an average of 25,558 tags per sample). More sequences were discarded from the V4F-V6R than the V6F-V6R dataset, indicating that the sequencing quality of the V4F-V6R dataset was inferior to that of the V6F-V6R. This difference in sequencing quality affected the α-diversity estimations, which will be discussed below. Secondly, we screened the chimeras with UCHIME. Because the sequencing of 101 bp from both ends could not sequence through the whole V4 to V6 region of the 16S rRNA, we linked each pair of tags with 30 Ns to allow screening of the chimeras. After this step, we acquired 263,127 tags from the V4F-V6R primer set (an average of 9,398 tags per sample) and 714,938 tags from the V6F-V6R primer set (an average of 25,533 tags per sample). Once again, many more chimeras were found with the V4F-V6R than the V6F-V6R dataset. This result is reasonable, as the V4 to V6 region (approximately 550 bp) is much longer than the V6 region (approximately 65 bp) and spans conservative sequences in the 16S rRNA, thus being more likely to form chimeras during the process of PCR amplification [17]. Finally, to unify the region and length of the tag, the same 60 bp sequence next to the V6R primer was extracted from both primer sets. To avoid the influence of different sequencing depths, we rarefied all samples to 5,000 tags for a consistent sequencing depth. The Good’s coverage of all samples with 5,000 tags was higher than 0.95 with 0.96 ± 0.005 (mean ± SEM) for samples from the V4F-V6R datasets and 0.98 ± 0.004 for the V6F-V6R datasets, indicating that the sequencing depth was sufficient for reliable analysis of these fecal microbial community samples. Based on these data, analyses including α-diversity (within-community diversity), β-diversity (between-communities diversity), microbial structure and biomarker determination were evaluated, as they are fundamental for microbiome research.

In addition to the quality filtering results, four external standards were sequenced simultaneously with each of the two libraries for a direct comparison of the sequencing quality. The external standards were samples with only one known cloned sequence as the PCR template, and the accuracy was checked at each base position. By comparing the sequencing results of the external standards with the known sequence, we could, to some extent, evaluate the sequencing quality of the library. All external standards were also filtered to remove ambiguous bases (N) and chimeras as above. As shown in Additional file 1: Figure S1, the proportion of sequences which have 100% identity with the external standard in the V6F-V6R library was higher than that of the V4F-V6R library (0.939 vs. 0.879, t-test, P < 0.001), while the proportion of error sequences was significantly lower in the V6F-V6R than the V4F-V6R library, indicating that the sequencing quality of the former was superior to that of the latter.

### α-diversity indices

**Figure 1:**
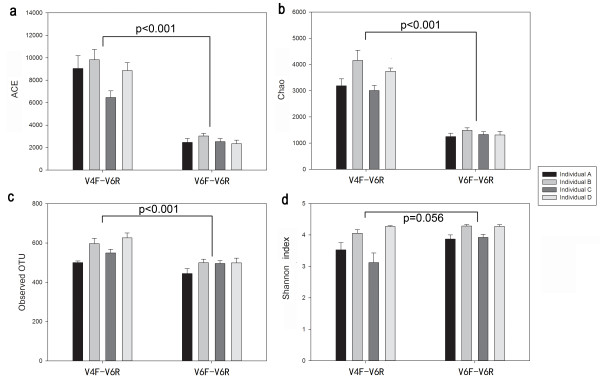
**α-diversity comparisons between the two datasets.** Mean values and 95% SEM are shown for each individual. Statistical analysis was performed using Mann-Whitney rank sum tests. Three species richness estimators, including **(a)** ACE **(b)** Chao and **(c)** number of OTUs, and one species evenness estimator, **(d)** Shannon’s diversity index, were included.

We evaluated four α-diversity indices, including the ACE, Chao, number of observed OTUs and Shannon index. The three species richness estimates (ACE, Chao, and observed OTUs) calculated using the V6 tag extracted from the V4F-V6R dataset were significantly higher than those calculated from the V6F-V6R dataset (P < 0.001) (Figure 1). It is reasonable to expect that all errors including PCR biases, PCR errors (mutations and chimeras), and sequencing errors could contribute to differences in the richness estimates. According to our quality control analysis, the sequencing quality of the V4F-V6R dataset was significantly inferior to that of the V6F-V6R dataset, and chimeras were also more prevalent in the former. These error sequences tend to be rare, as the same error is unlikely to occur multiple times [18,19]. Because species richness estimators such as ACE and Chao mainly depend on the number of rare OTUs (for example, the Chao is calculated only with the number of singletons and doubletons), the V6 tag from the V4F-V6R dataset, which contained more errors, obtained significantly higher richness estimates. The fact that each library was only sequenced once reduced the statistical power for evaluating the adverse effects of sequencing errors.

Not surprisingly, the meta-analysis of species richness was significantly biased by the data source. For example, if we chose sequences from the V4F-V6R dataset for individuals A and B and sequences from the V6F-V6R dataset for individuals C and D (simulating a situation where sequences are obtained by various methods from individuals A and B in one experiment and from individuals C and D in another experiment prior to combination of the data), then A and B had much higher species richness estimates than C and D, a result which actually reflects differences in the generation of the two datasets (sequencing and PCR errors) rather than the diversity of the samples. Although we used the same HiSeq 2000 instrument for both of the datasets, the sequencing quality of the two sequencing batches was obviously different. For those datasets preserved in databases, individuals using various 454 and Illumina instruments obtained different sequencing qualities, a factor which is problematic for meta-analysis of richness estimates.

In contrast, Shannon’s diversity index showed no significant difference between the two datasets (3.77 ± 0.10 for V4F-V6R versus 4.06 ± 0.06 for V6F-V6R, P = 0.056), indicating that this index was more stable than the richness estimators and more reliable for comparison across various studies. In addition, we randomly changed the bases of these sequences to simulate sequencing errors rates of 0.1% and 1% to evaluate the effect of sequencing errors on the estimation of alpha indices. The results demonstrated that at an error rate of 0.1%, all indices including the richness estimates and Shannon index were hardly influenced (One Way ANOVA on ranks, P < 0.05, Dunn’s test for pair-wise comparisons between 0% error rate and 0.1% error rate, P > 0.05), but raising the error rate to 1% inflated the species richness estimates significantly (One Way ANOVA on ranks, P < 0.05, Dunn’s test for pair-wise comparisons between 0% error rate and 1% error rate: ACE, 546 vs. 2435, P < 0.05; Chao1, 886 vs. 3680, P < 0.05; observed species, 285 vs. 577, P < 0.05). By comparison, although the Shannon index was also inflated (5.37 vs. 5.90, P < 0.05), the extent of inflation was much smaller than that of the species richness estimators, and no significant differences were observed between the two datasets (Additional file 1: Figure S3). The explanation for this result is that Shannon diversity index depends more on highly abundant OTUs compared to species richness estimates [20], is consequently less sensitive to sequencing errors and was therefore able to produce similar values for both of the datasets in the present study. In support of this theory, we found in a recent study [20] that Shannon diversity index of freshwater and marine sediments were comparable across multiple studies.

### PCA using the Jaccard distances

**Figure 2:**
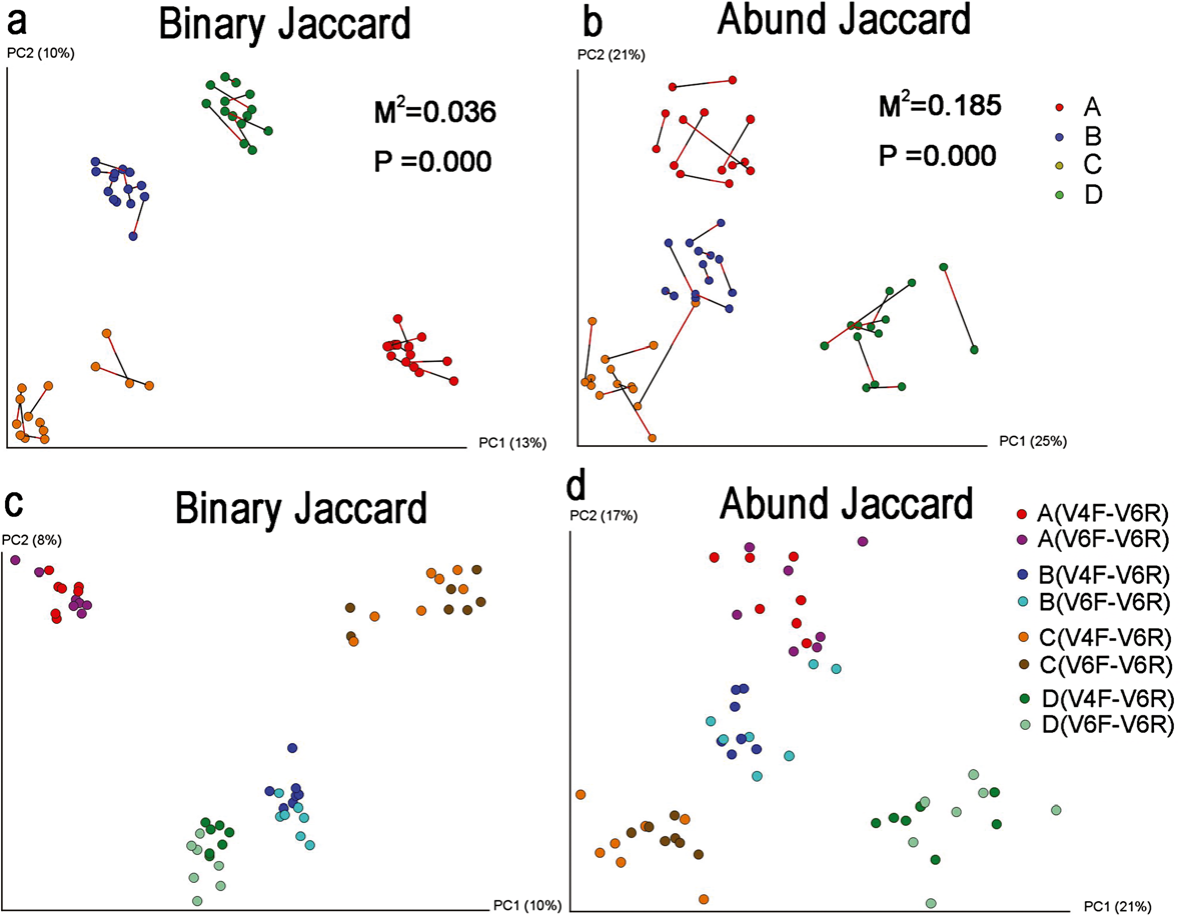
**Principal component analysis of binary and abundance-weighted Jaccard distances between samples. (a** and **b)** Procrustes analysis of PCA results based on binary **(a)** or abundance-weighted **(b)** Jaccard distances of the two datasets. Points linked with bars were obtained from the same individual but from two different datasets. **(c)** and **(d)** Two datasets were combined for meta-analysis based on binary **(c)** or abundance-weighted **(d)** Jaccard distance.

We next compared the two datasets in terms of β-diversity obtained using Principal Component Analysis (PCA) with Jaccard distances (Figure 2a, b). The rationale for using the Jaccard, rather than the phylogenetic-based UniFrac, distances is that the V6 tag is very short with high variability, leading to a relatively lower resolution of the UniFrac distance after alignment and filtering of unmatched sequences. Procrustes analysis illustrates two PCA analyses in one plot, transforming one of the coordinate sets by rotating, scaling, and translating it to minimize the distances between two corresponding points of the same sample. The results of the two datasets (the V6 fragment extracted from two different PCR and sequencing runs) were in accordance with each other based on the abundance-weighted and binary Jaccard distances (p = 0.000), with obvious clustering of samples from each individual.

Subsequently, we combined all sequences from these two datasets to simulate a meta-analysis (Figure 2c, d). PCA of both binary and abundance-weighted Jaccard distances demonstrated that the pooled analysis resulted in almost the same clustering as the single dataset, and the points from the two data sources could hardly be differentiated, suggesting that meta-analysis using the Jaccard distance is relatively stable across different PCR and sequencing runs. To further understand the effect of sequencing errors on PCA, we performed procrustes analysis with the original datasets vs. datasets with simulated base error rates of 1% (Additional file 1: Figure S4). All pair-wise comparisons show that sequencing errors did not greatly affect the PCA based on the Jaccard distance, in support of our conclusions detailed above.

### Microbial composition and biomarker determination

**Figure 3:**
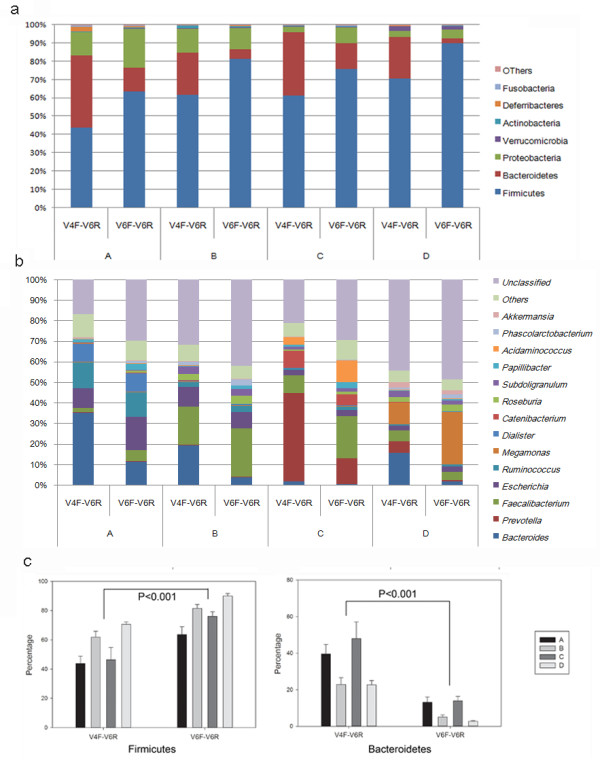
**Microbial structure at phylum and genus level. (a)** Microbial structures of each individual determined at the phylum level by the two primer sets. **(b)** Microbial structures of each individual determined at the genus level by the two primer sets. **(c)** Relative abundance of Firmicutes and Bacteroidetes determined by the two primer sets.

The two datasets showed significantly different community structures (Figure 3a). Although the gut flora of all subjects consisted primarily of Firmicutes, Bacteroidetes and Proteobacteria, the relative abundance of these microbes varied significantly. Compared to the V6F-V6R dataset, the V4F-V6R dataset identified higher levels of Bacteroidetes and lower levels of Firmicutes (Figure 3c). Interestingly, the categories of genera identified by the two primer sets were similar to each other, while the relative abundance of the genera differed (Figure 3b). We suggest that both the primer bias and sequencing errors contributed to these differences, but the former may have contributed more because sequencing errors usually occur at a very low frequency and do little to change the overall relative abundance. Several studies have compared microbial community structures using different primer sets [11,21]. These studies usually found significant primer biases in the evaluation of microbial ecology. However, here we demonstrated for the first time that PCA using the Jaccard distance was minimally affected by primer bias and differences in sequencing quality, suggesting the feasibility of performing meta-analysis for sequences obtained from different sources.

**Figure 4:**
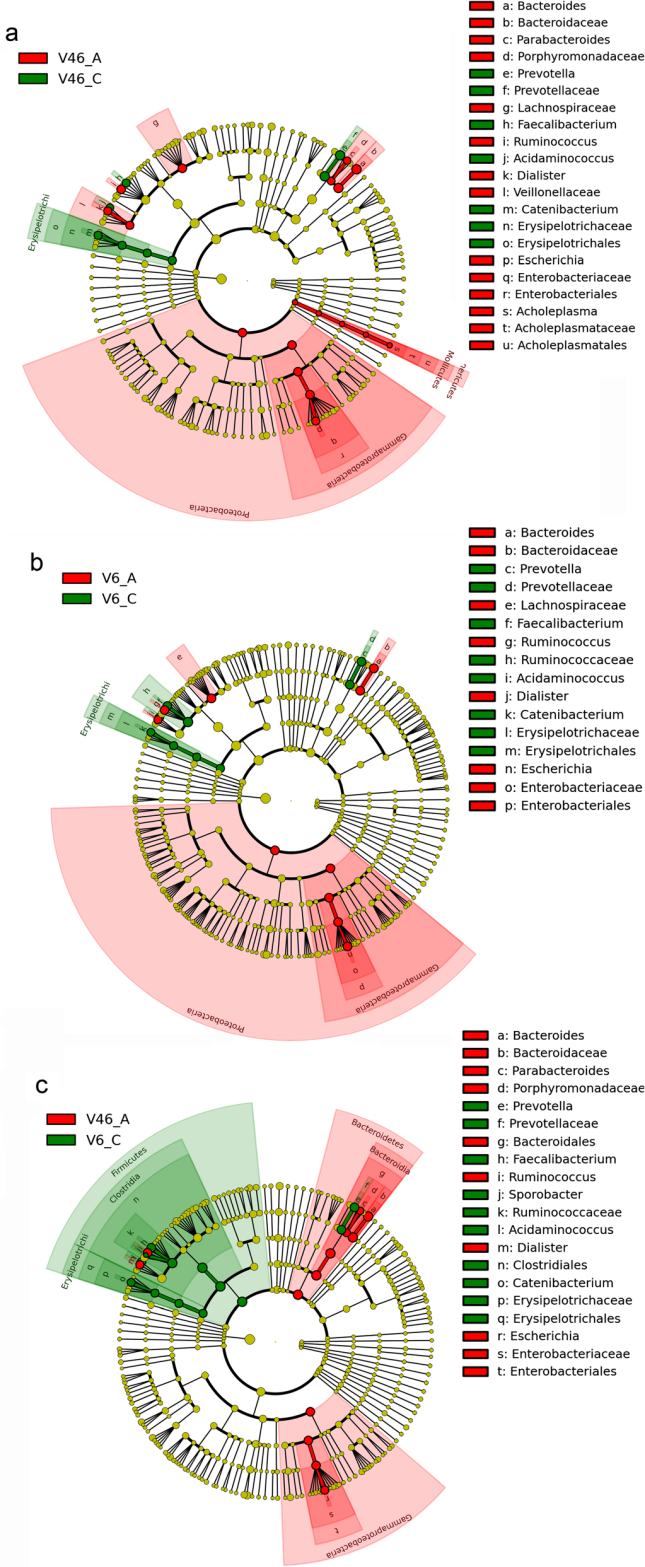
**LEfSe comparison of microbial communities between individuals A and C with different data sources. (a)** Individual A and C are both from V46 library. **(b)** Individual A and C are both from V6 library. **(c)** Individual A is from V46 library and Individual C is from V6 library.

We used LEfSe for the quantitative analysis of biomarkers within different groups (Figure 4 and Additional file 1: Figure S2). This method was designed to analyze data in which the number of species is much higher than the number of samples and to provide biological class explanations to establish statistical significance, biological consistency, and effect-size estimation of predicted biomarkers [16]. To simulate a simple meta-analysis, we compared the microbiomes of four individuals two at a time (e.g., A vs. C and B vs. D). The results demonstrated that when the data from the two individuals came from the same dataset, their biomarkers were generally similar. For example, the biomarkers identified using the V4F-V6R dataset for individuals A and C were similar to those obtained using the V6F-V6R dataset (Proteobacteria, *Bacteroides*, Lachnospiraceae, and Enterobacteriaceae for individual A and *Prevotella*, *Faecalibacterium*, and Erysipelotrichaceae for individual C) (Figure 4a, b). However, when we analyzed the microbiome data of individual A from the V4F-V6R dataset and the data of individual C from the V6F-V6R dataset, the Firmicutes phylum was identified for individual C, and Proteobacteria was no longer identified as a biomarker for individual A (Figure 4c). Surprisingly, when we analyzed the microbiome data for individual A from the V6F-V6R dataset and the data for individual C from the V4F-V6R dataset, no biomarkers were identified for the two groups (not shown in Figure 4, as no biomarkers were identified). A similar situation occurred when analyzing the data from individuals B and D, as there were no biomarkers identified when the V6F-V6R dataset was used for individual B and the V4F-V6R dataset was used for individual D (Additional file 1: Figure S2). Taken together, these results suggest that while similar biomarkers can be obtained even when different primer sets and sequencing batches are used, meta-analysis should be performed cautiously when using data obtained from different sources.

## Conclusions

For the purposes of meta-analysis, PCA using both the binary and abundance-weighted Jaccard distance is reliable, and Shannon diversity index is also relatively stable across different studies. However, the richness estimators, especially those depending primarily on rare tags (e.g., Chao and ACE) are significantly affected by the experimental procedures unique to individual studies. The community structure, especially the relative abundance, also varies significantly between different datasets. Biomarkers between different groups are comparable between multiple experiments if the input data for the LEfSe analysis is obtained from a single experiment, but meta-analyses using combined datasets should be performed cautiously. In the present study, we only take into account primer bias and sequencing quality, and their effect on microbiota analyses from combined studies, variations in the experimental procedures of different laboratories could also affect the meta-analyses. Additional studies verifying the PCR conditions, particularly the enzyme system, DNA extraction, DNA storage effect, etc., are needed in future.

## Competing interest

The authors declare no competing financial interests.

## Authors’ contributions

YH, XTJ and HWZ conceived of the study. BJZ and GHD performed the experiments. YH, XTJ and HZ analyzed the data. YH and HWZ wrote the manuscript. All authors read and approved the final manuscript.

## Supplementary Material

Additional file 1: Figure S1 Using external standards to compare the sequencing qualities between the two libraries. The identity with external standard sequence is split into four groups and we calculated the proportion of sequences in each sequencing batch fall into each group. **Figure S2.** LEfSe comparison of microbial communities between individuals B and D with different data sources. **Figure S3.** Alpha diversity index calculated from the V6F-V6R and V4F-V6R datasets at error rates of 0%, 0.1% and 1%. **Figure S4.** Procrustes analysis of datasets from the two libraries and error rates. Click here for file
