# Supplementary material for: Comparison of microbial diversity determined with the same variable tag sequence extracted from two different PCR amplicons
Source: BMC Microbiol. 2013 Sep 14;13:208. doi: 10.1186/1471-2180-13-208 (PMC3848352; doi:10.1186/1471-2180-13-208)
Supplement: Additional file 1: Figure S1 — Using external standards to compare the sequencing qualities between the two libraries. The identity with external standard sequence is split into four groups and we calculated the proportion of sequences in each sequencing batch fall into each group. Figure S2. LEfSe comparison of microbial communities between individuals B and D with different data sources. Figure S3. Alpha diversity index calculated from the V6F-V6R and V4F-V6R datasets at error rates of 0%, 0.1% and 1%. Figure S4. Procrustes analysis of datasets from the two libraries and error rates. [file 1471-2180-13-208-S1.doc]

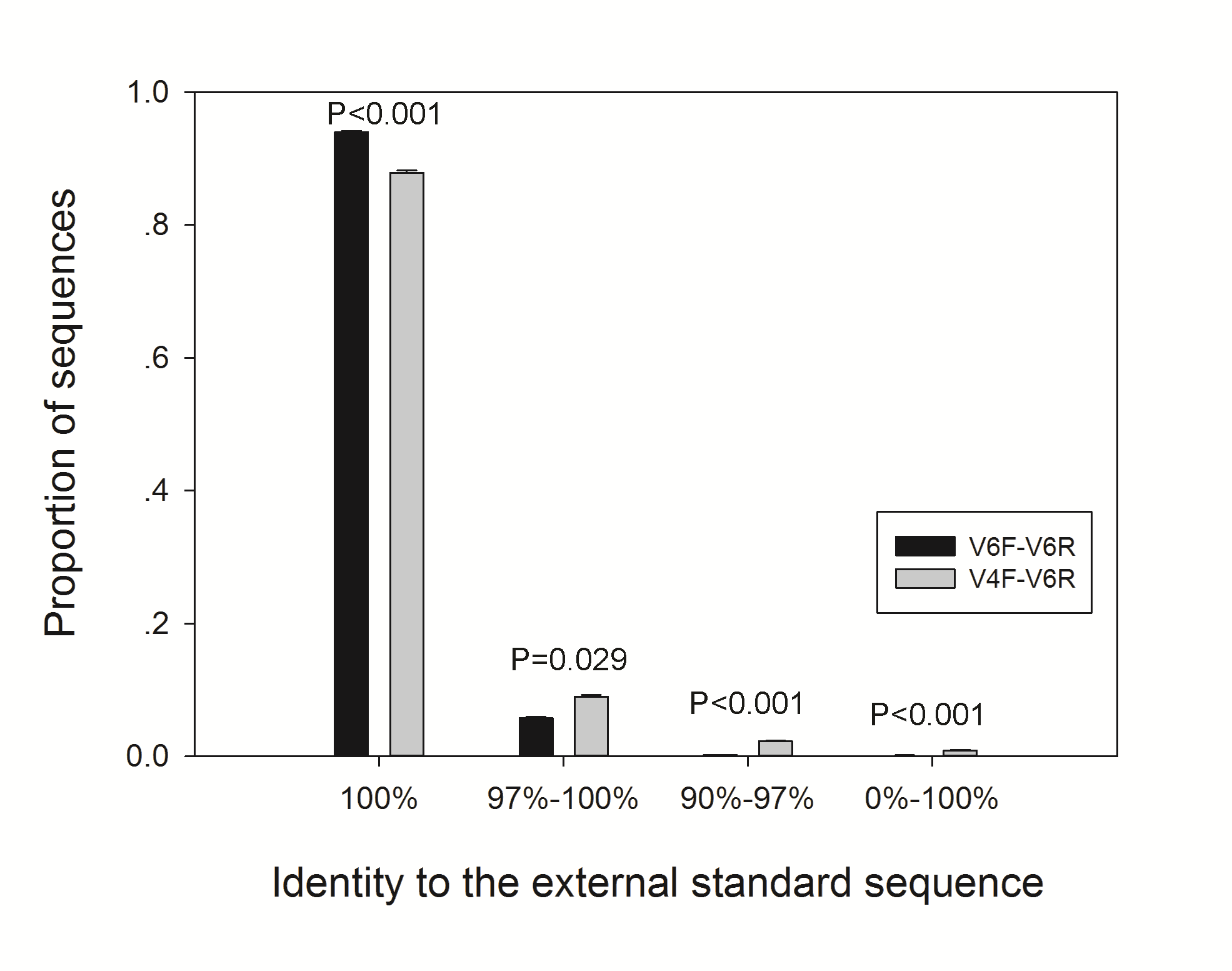


Fig. S1 Using external standards to compare the sequencing qualities between the two libraries. The identity with external standard sequence is split into four groups and we calculated the proportion of sequences in each sequencing batch fall into each group.


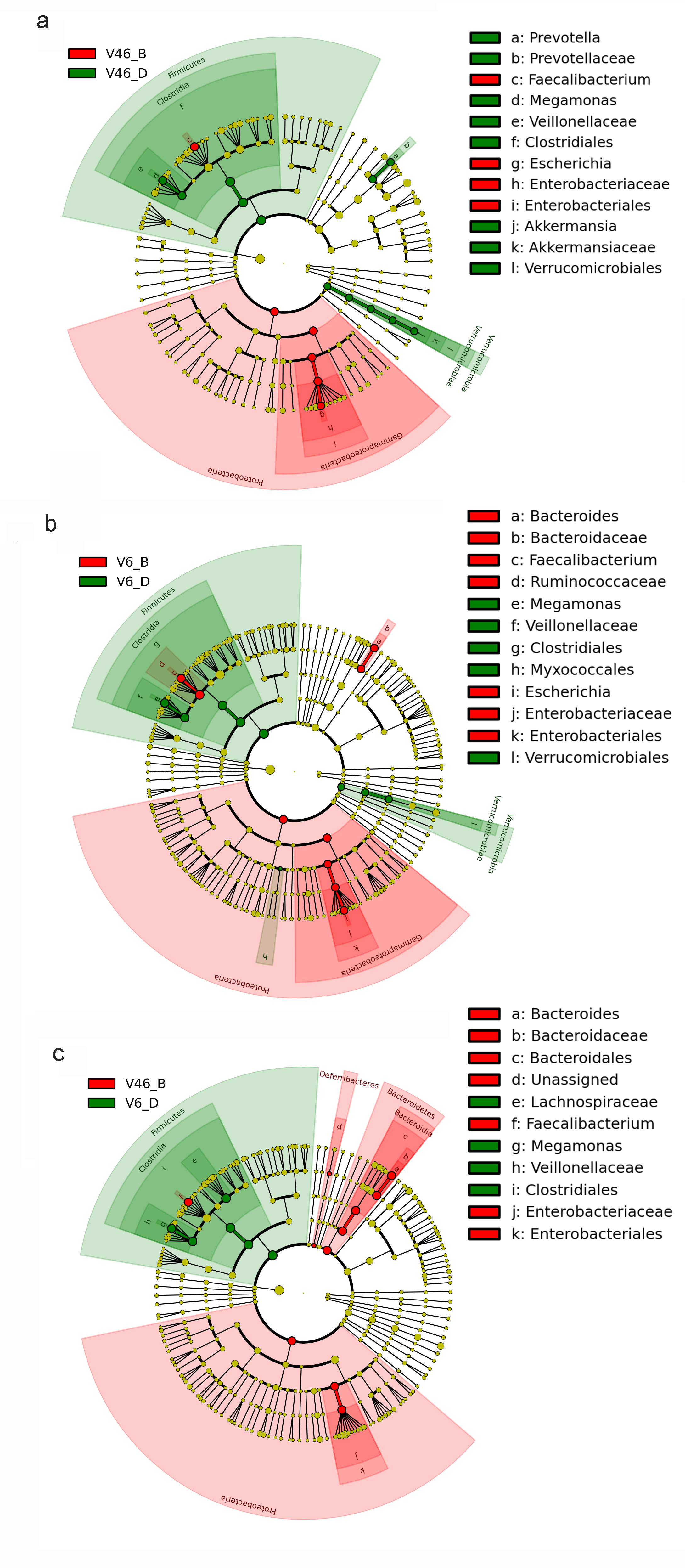


Fig. S2 LEfSe comparison of microbial communities between individuals B and D with different data sources.


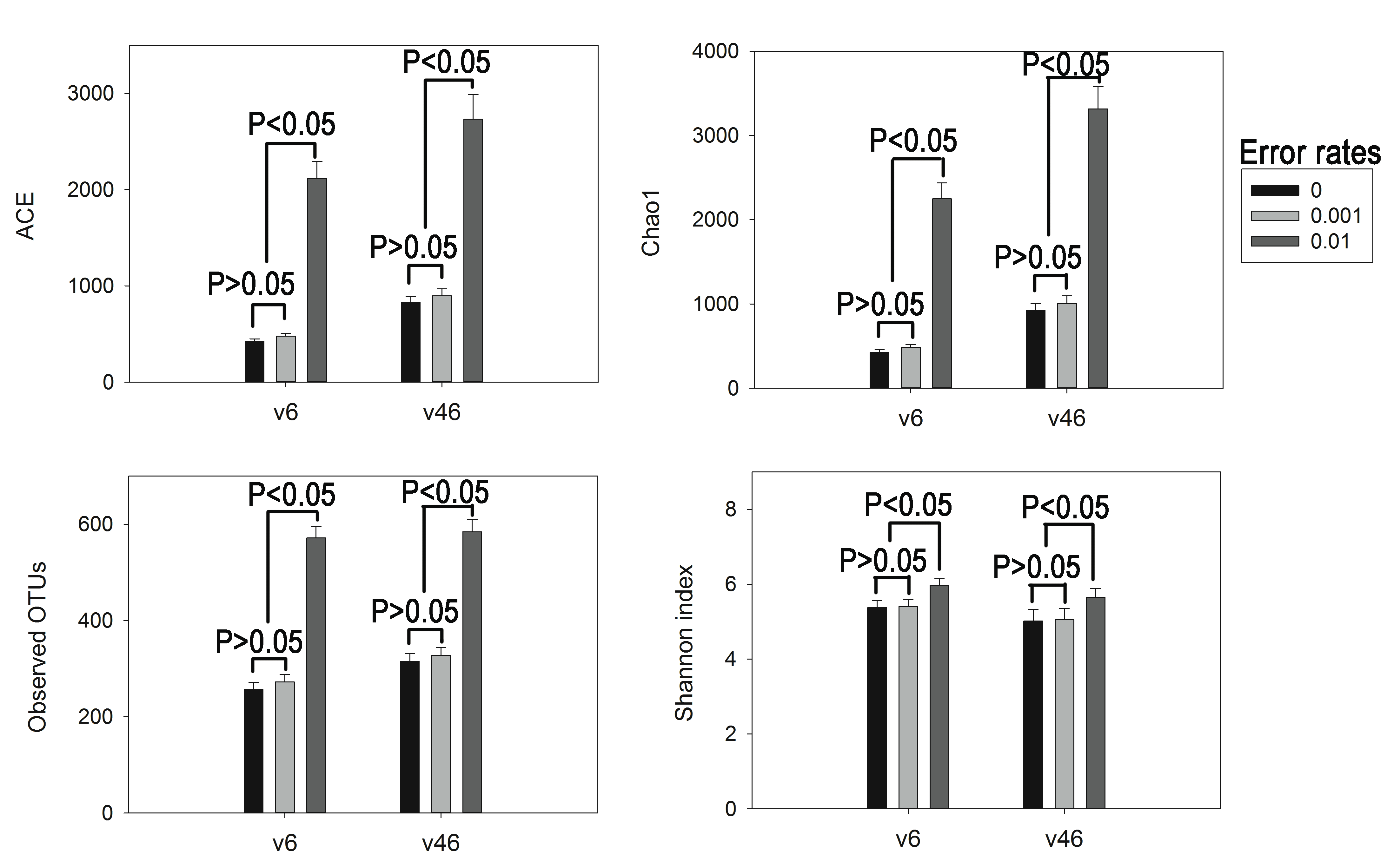


Fig. S3 Alpha diversity index calculated from the V6F-V6R and V4F-V6R datasets at error rates of 0%, 0.1% and 1%.


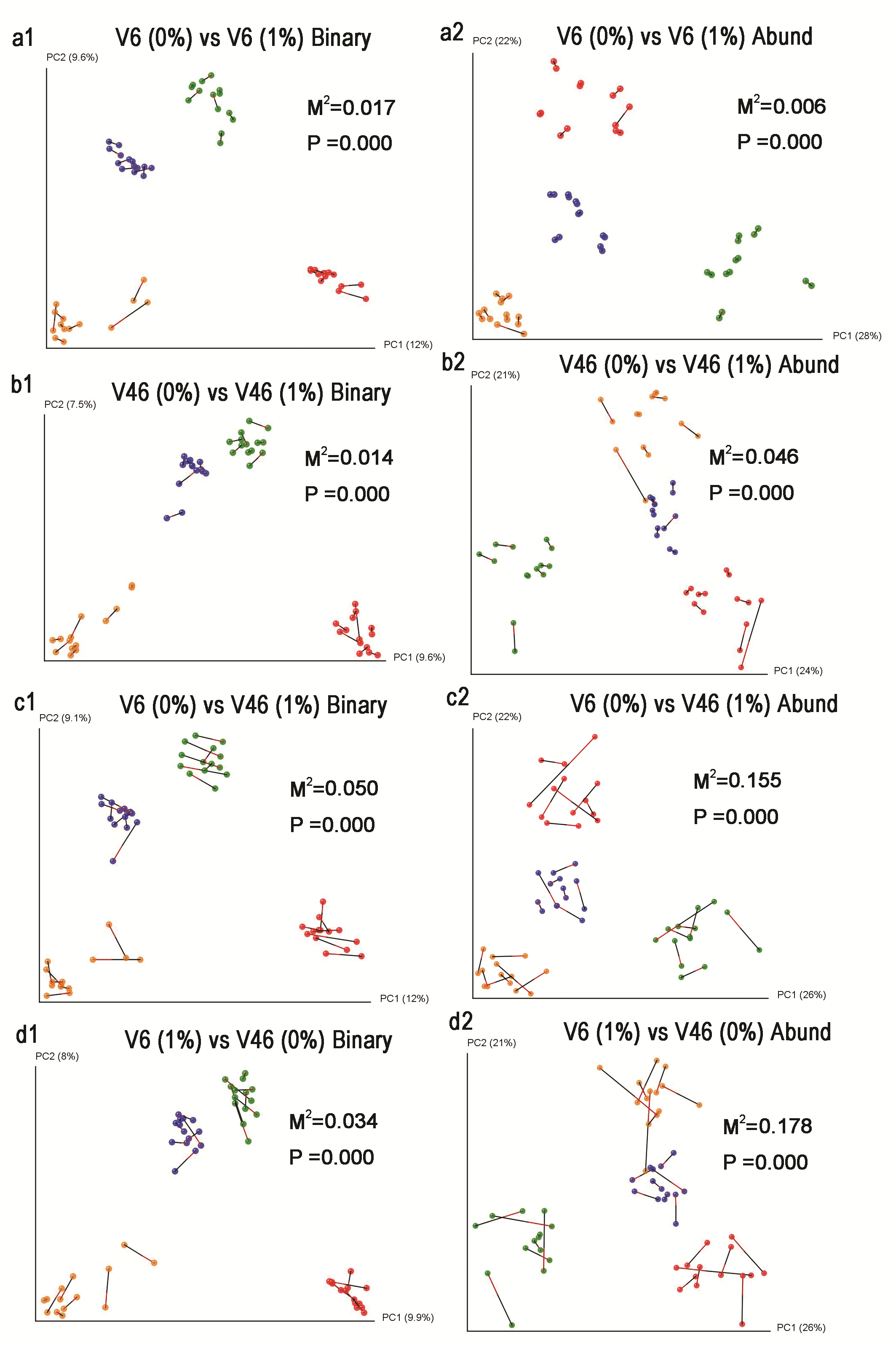


Fig. S4 Procrustes analysis of datasets from the two libraries and error rates.
